# Lung Cancer in Combined Pulmonary Fibrosis and Emphysema: A Systematic Review and Meta-Analysis

**DOI:** 10.1371/journal.pone.0161437

**Published:** 2016-09-12

**Authors:** Hyun Jung Koo, Kyung-Hyun Do, Jung Bok Lee, Sania Alblushi, Sang Min Lee

**Affiliations:** 1 Department of Radiology and Research Institute of Radiology, University of Ulsan College of Medicine, Asan Medical Center, Seoul, Korea; 2 Clinical Epidemiology and Biostatistics, University of Ulsan College of Medicine, Asan Medical Center, Seoul, Korea; 3 Maternity and Children’s Hospital, Dammam, Saudi Arabia; Imperial College London, UNITED KINGDOM

## Abstract

**Purpose:**

Patients with combined pulmonary fibrosis and emphysema (CPFE) have been suggested to have an increased risk of lung cancer. We conducted a systematic review of all published data and performed a meta-analysis to define the characteristics of lung cancer that develops in CPFE.

**Method:**

We searched Pubmed, Embase, and Cochrane to find original articles about lung cancer and CPFE published prior to September 2015. All titles/abstracts were reviewed by two radiologists to identify articles that used predefined selection criteria. Summary estimates were generated using a random-effect model and odds ratios (ORs) to develop squamous cell carcinoma (SqCC) were calculated. Kaplan–Meier survival curves were obtained for the survival of patients with CPFE and non-CPFE.

**Results:**

Nine original articles that assessed 620 patients were included in this review. In the pooled data, patients were older age (70.4 years), almost all were heavy smokers (53.5 pack years), and males were predominant (92.6%). SqCC was the most common type (42.3%), followed by adenocarcinoma (34.4%). Compared with lung cancer population with an otherwise normal lung, the OR to develop SqCC in CPFE was 9.06 (95% CI, 6.08–13.5). The ORs in CPFE compared with lung cancers that developed in lungs with fibrosis or emphysema were also higher. The median survival for CPFE patients with lung cancer (19.5 months) was significantly shorter than in non-CPFE (53.1 months).

**Conclusions:**

Lung cancer in CPFE, most commonly SqCC, presents in elderly heavy smokers with a male predominance. The median survival for CPFE patients with lung cancer is 19.5 months.

## Introduction

Patients with idiopathic pulmonary fibrosis (IPF) or chronic obstructive pulmonary disease (COPD) are at an increased risk of developing lung cancer, especially in the case of male smokers [1–3]. The treatment of lung cancer in patients with IPF or COPD is challenging, because vigorous treatments, including surgery, radiation therapy, and chemotherapy, may induce iatrogenic acute exacerbation or pneumonia that may result in lethal complications and mortality.

The coexistence of pulmonary fibrosis and emphysema has been suggested since 1990 [4]. More recently, combined pulmonary fibrosis and emphysema (CPFE) with upper lobe emphysema and lower lobe fibrosis of the lung has been recognized as an unique entity [5, 6]. Several previous studies have suggested that patients with CPFE can present distinct clinical characteristics that are associated with different outcomes [7, 8]. CPFE has also been associated with a high risk of developing lung cancer (up to 46%) [7], and is more prevalent than fibrosis in lung cancer patients [9]. In two previous studies of patients with lung cancer and CPFE, adenocarcinoma was the most common type of cancer (43%), followed by squamous cell carcinoma (35%) [9, 10]; however, other studies have shown that squamous cell carcinoma is the most common type of cancer in CPFE patients [3, 7, 11, 12]. The overall survival of CPFE patients with lung cancer has also been reported in several studies [9–11, 13, 14], and the results have varied. Although lung cancer patients without CPFE exhibit better survival outcomes compared with patients with CPFE [9], CPFE may not be an independent prognostic factor in lung cancer patients with idiopathic interstitial pneumonia [14].

Previous studies should be re-evaluated to clarify their inclusion criteria for CPFE, as it has not had a standardized definition and the characteristics of lung cancer in CPFE have not yet been fully evaluated. Herein, we conducted a systematic review and meta-analysis of all available studies to determine the risk of lung cancer in CPFE, to describe the histopathology of lung cancer in CPFE and to assess mortality after lung cancer diagnosis.

## Materials and Methods

This present study was conducted according to the guidelines suggested by the Meta-analysis of Observational Studies in Epidemiology group based on the Preferred Reporting Items for Systematic Reviews and Meta-Analyses (PRISMA) Statement (S1 File) [15]. We searched the PUBMED, EMBASE, and COCHRANE databases to identify all observational studies of patients with CPFE (S1 Table). Because CPFE had been termed combined cryptogenic fibrosing alveolitis and emphysema [4], the following search terms were used: “pulmonary fibrosis” AND “emphysema” AND [“fibrosis” OR “fibroses” OR “fibrosing” OR “alveolitis” OR “alveolitides”] AND [“combine*” OR “cryptogen*”]. The start date for the search was 2005, as the first article to describe the criteria for CPFE as a distinct entity was published in 2005 [5]. The literature search was restricted to English-language articles. The last search was performed on October 7, 2015, and a manual search was performed.

Inclusion criteria were as follows: (1) population: cohort or case-control study of lung cancer patients with a group of patients with CPFE; (2) intervention: surgical resection or pathologically confirmed cancer; (3) comparison: cancer patients without CPFE, such as cancers that develop in a normal lung (no abnormality except for the presence of the lung cancer) or a lung with fibrosis or emphysema alone; and (4) outcome: median survival time and survival curves that include 1- to 3- or 5-year survival rates. Exclusion criteria were as follows: (1) case reports; (2) review articles, letters, comments, or conference proceedings; (3) studies of subjects other than lung cancer patients with CPFE; (4) studies with an overlapping population; and (5) non-English full text. In nine patients of a study [11], the diagnosis of lung cancer could not be pathologically confirmed due to failure of diagnostic procedure, patients’ pulmonary function alteration, and/or poor general condition. For the patients, lung cancer diagnosis used the presence of a persistent spiculated or heterogenous lung mass, the growth of the lesion, and a strong hyper-metabolism of the lesion on 18-fluoro-deoxy glucose positron emission tomography scan, along with the attempts to rule out infection. We have included the nine patients to evaluate the risk of cancer in CPFE; however, the patients were excluded when we analyze the pathologic types of cancers in CPFE.

### Data review

To identify relevant studies, two radiologists (K.H.D. and H.J.K.) independently reviewed all titles and abstracts. A full-text review was carried out using inclusion and exclusion criteria. The two reviewers independently retrieved information about the study design, year, country, number of patients, overall survival, and survival curves. Disagreements between the two reviewers were resolved by consensus. In all but two of the included articles, the definition of CPFE was based on a previous article [5]: (a) upper lobe emphysema defined as an area of reduced attenuation compared with the normal lung, no wall or marginated by a thin wall of less than 1 mm, and/or multiple blebs/bullae with an upper lung zone predominance; and (b) diffuse pulmonary fibrosis in the lower lobes on chest high-resolution CT. Pulmonary fibrosis was defined as lung regions of irregular and reticular opacities with a subpleural and basal predominance, honeycombing, parenchymal distortion or retraction, and bronchiectasis or bronchioloectasis. In one of the two exceptional studies, CPFE was independently diagnosed by two pulmonologists based on the coexistence of emphysema of grade 2 or more (% low attenuation area [%LAA] ≥25%) and significant pulmonary fibrosis in patients with parenchymal lung disease [7]. The emphysema grade was calculated as the sum of the scores for the percentage of involved tissue of a low attenuation area in six lung fields: score 0, %LAA < 5%; score 1, 5% ≤ %LAA < 25%; score 2, 25% ≤ %LAA < 50%; score 3, 50% ≤ %LAA < 75%; and score 4, 75% ≤ %LAA. Grade 2 included total scores of 7–12. All patients in the study cohort had upper lung field dominant emphysema with lower lung field dominant lung fibrosis. In one study [16], diagnostic criteria for IPF and emphysema were used [8, 17], and patients who met both of these criteria were included as CPFE cases. Lung cancer staging was based on the Tumor, Node, Metastasis system, 7^th^ edition [18]. Pathological stages were gathered if they were available. Additionally, if there were patients with lung cancer that could not be confirmed pathologically because of deteriorating pulmonary function, the failure or complication of diagnostic procedures, or a poor general condition leading to the refusal of a procedure, clinical stages were used instead. The included studies were appraised using a modified Downs and Black checklist (S2 and S3 Tables)

### Statistical Analysis

Pooled data that was produced using the inverse variance method for calculating each weight, along with the calculated pooled proportion and its 95% confidence interval (CI) were obtained. Forest plots were generated by using random-effects models. Study heterogeneity was assessed using the *χ*^2^ test, and significant heterogeneity was defined as a *P-*value <0.10. Values of I^2^ > 50% were deemed to indicate substantial heterogeneity. Publication bias was not evaluated because of the small number of included studies in this meta-analysis. Summary survival curve data were obtained using five studies from which Kaplan–Meier survival curves were available [19]. Cancer stages between the CPFE and non-CPFE groups were compared using the *χ*^2^ test. All statistical analyses were performed using Comprehensive Meta-analysis (CMA) software, version 2.0 (CMA 2.0, Biostat Inc., Englewood, NJ) and R version 3.0.2 (The R Foundation for Statistical Computing, Vienna, Austria).

## Results

**Fig 1:**
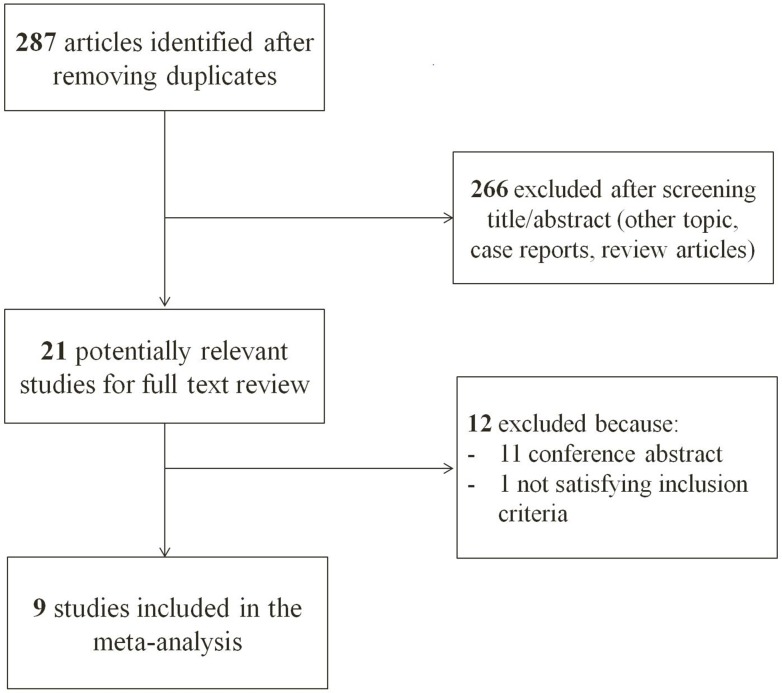
A flow diagram of the study.

After removing duplicate data between the three databases, 287 articles were identified from the literature search (Fig 1). After excluding 266 papers with non-relevant titles and/or abstracts, 21 articles were retrieved for full-text review. Among these articles, 11 were conference abstracts and 1 study did not fulfill the inclusion criteria as the chemotherapy induced interstitial lung disease other than lung cancer. Ultimately, 9 original articles—8 cohort studies and 1 case-control study—were included for review. The major characteristics of the studies are listed in Table 1. Most studies were carried out in Japan, apart from two studies conducted in France and Korea. Six studies consisted of lung cancer cohorts, and among them, 2 studies included patients who presented with surgically resected lung cancer. Two studies were conducted using CPFE cohorts and identify the incidence of lung cancer. Remained 1 study was case-control study comparing CPFE versus non-CPFE. In the 6 cohort studies, 4 studies compared four groups of patients with lung cancer that developed in a normal lung or lungs with fibrosis, emphysema, or CPFE. In 2 studies, patients with CPFE were compared with non-CPFE patients. Kaplan–Meier curves for survival analysis were shown in 5 of the studies. All studies reported age, gender, and smoking history, including pack years. The clinical characteristics of the lung cancer patients with CPFE at the time of lung cancer diagnosis are shown in the Table 2. In the pooled data, patients were older (70.4 years), almost all were heavy smokers (98.6%, 53.5 pack years), and showed a male predominance (92.6%). Squamous cell carcinoma was the most common type of cancer (42.3%), followed by adenocarcinoma (34.4%) (Table 3).

**Table 1:** Case-control and cohort studies on CPFE and cancer risk in the meta-analysis.

Study/year	Country	Study design	Number of patients			Enrolled period	Group	Adjusted variables for regression model	Notes for conclusions	Quality assessment score (24)^a^
			Cancer	CPFE	CPFE with cancer					
**Kumagai et al/2014**	Japan	Single center, cohort	365	20	20	2007–2012	Normal/Fibrosis/Emphysema/CPFE	OS	Complete resection group. CPFE was an independent prognostic factor for DFS and OS.	18
**Fugiwara et al/2013**	Japan	Single center, cohort	274	36	36	2003–2011	Normal/Fibrosis/Emphysema/CPFE		The prevalence of SqCC of the peripheral areas was higher in the CPFE patients.	11
**Mimae et al/2015**	Japan	Multicenter, cohort	2333	157	157	2008–2010	CPFE vs. non-CPFE	DFS, OS	Lung cancer, resection group. CPFE with lung cancer patients showed poor prognoses regardless of good PFT results.	18
**Minegishi et al/2014**	Japan	Single center, cohort	1536	88	88	1998–2011	CPFE vs. non-CPFE	OS	CPFE was not an independent prognostic factor of lung cancer in IIP patients.	18
**Usui et al/2011**	Japan	Singe center, cohort	1143	101	101	2002–2009	Normal/Fibrosis/Emphysema/CPFE	OS	In lung cancer patients, CPFE was more prevalent than lung fibrosis. CPFE patients showed a poor prognosis.	18
**Girard et al/2014**	France	Multi-center, cohort	47	322	47	2003–2012	CPFE		Poor prognosis	15
**Fukui et al/2014**	Japan	Single center, cohort	1507	137	137	2008–2013	Normal/Fibrosis/Emphysema/CPFE	OS	The postoperative outcome of CPFE patients was poor.	18
**Kwak et al/2013**	Korea	Single center, case-control	12	48	12	2000–2011	CPFE/fibrosis/emphysema	Lung cancer risk, mortality	CPFE had a higher risk of lung cancer or death compared with the emphysema group.	16
**Kitaguchi et al/2010**	Japan	Single center, cohort	22	47	22	2004–2007	CPFE	Lung cancer risk, PFT	High prevalence of lung cancer	15

CPFE, combined pulmonary fibrosis and emphysema; DFS, disease-free survival; IIP, idiopathic interstitial pneumonia; OS, overall survival; PFT, pulmonary function test; SqCC, squamous cell carcinoma.

^a^Results of the risk of bias assessment using the Downs and Black quality assessment scale, total 24 points (S2 and S3 Tables)

**Table 2:** Clinical characteristics of the patients with CPFE and lung cancer.

Study/year	Age	Male %	Smoking%	PY	VC%	FEV1%	FEV1/FVC%	DLCO%
**Fugiwara et al/2013**	69.0	94.4	97.2	54.0	-	-	-	-
**Fukui et al/2014**	70.9	90.5	-	58.0	98.6	71.0	-	44.0
**Girard et al/2014**	68.0	97.9	100	47.0	-	74.0	78.0	-
**Kitaguchi et al/2010**^a^	70.3	97.9	-	58.7	94.7	79.0	71.8	39.6
**Kumagai et al/2014**	76.0^b^	90.5	100	-	96.4	81.7	67.7	-
**Kwak et al/2013**^a^	66.6	100	100	43.2	-	95.0	75.7	-
**Mimae et al/2015**	73.0	92.4	100	-	98.0	71.5	-	-
**Minegishi et al/2014**	69.6	92.0	-	57.5	88.2	89.3	75.0	57.8
**Usui et al/2011**	70.0^b^	95.0	100	51.5	-	-	-	-
**Pooled Data**	70.4	92.6	98.6	53.5	95.2	79.4	73.1	46.9

CPFE, combined pulmonary fibrosis and emphysema; DLCO, diffusing capacity of lung for carbon monoxide; FEV1, forced expiratory volume in one second; FVC, forced vital capacity; PY, pack years; VC, vital capacity.

^a^In two studies, the value was demonstrated in the total CPFE population, including patients both with and without lung cancer.

^b^Studies that present age as a median and range.

**Table 3:** Pathologic types of lung cancer in the patients with CPFE.

Study/year	No. of pts with CPFE and lung cancer	Adeno %	SqCC %	Others %
**Fugiwara et al/2013**	36	30.6	52.8	16.7
**Fukui et al/2014**	137	40.1	46.7	13.1
**Girard et al/2014**	47	29.8	36.2	34.0
**Kitaguchi et al/2010**	22	31.8	54.5	13.6
**Kumagai et al/2014**	20	45.0	40.0	15.0
**Kwak et al/2013**	12	8.3	41.7	50.0
**Mimae et al/2015**	157	36.3	47.8	15.9
**Minegishi et al/2014**	88	21.6	36.4	42.0
**Usui et al/2011**	101	45.5	30.7	24.8
**Pooled Data**	620	34.4	42.3	23.4

CPFE, combined pulmonary fibrosis and emphysema; Adeno, adenocarcinoma; SqCC, squamous cell carcinoma; pts, patients.

**Fig 2:**
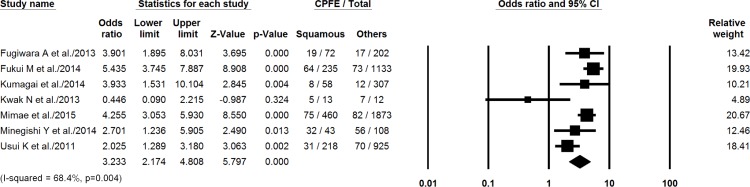
Summed odds ratios for the proportion of squamous cell carcinoma in patients with CPFE compared with that in non-CPFE patients.

**Fig 3:**
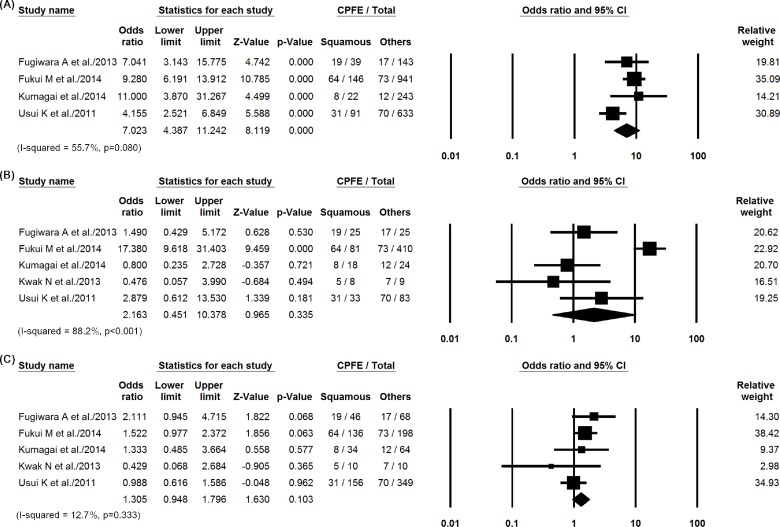
Summed odds ratios for the proportion of squamous cell carcinoma in patients with CPFE compared with (A) normal lungs or those with (B) fibrosis or (C) emphysema.

In the studies that we included, pooled data showing the characteristics of the comparison groups are presented in Table 4. The summed OR for the proportion of squamous cell carcinoma in patients with CPFE compared with that in non-CPFE patients was 3.23 (95% CI, 2.17–4.81, *P* < 0.001) (Fig 2). Compared with the lung cancer population with an otherwise normal lung, the odds ratio to develop squamous cell carcinoma in the CPFE group was 7.02 (95% CI, 4.39–11.2, *P* < 0.001). Fig 3 shows forest plots for the following comparisons: CPFE versus normal lung, CPFE versus fibrosis, and CPFE versus emphysema. I-squared values for the comparisons were 55.7% (*P* = 0.08), 88.2% (*P* < 0.001) and 12.7% (*P* = 0.33). The ORs in the CPFE group were higher compared with lung cancers developing in lungs with fibrosis (OR, 2.16, 95% CI, 0.45–10.38, *P* = 0.34) or emphysema (OR, 1.31, 95% CI, 0.95–1.80, *P* = 0.10) alone, without statistical significance. The summed OR for the proportion of adenocarcinoma in patients with CPFE compared with that in non-CPFE were demonstrated in S1 and S2 Figs. Apart from 37 patients without staging information and 98 patients with small-cell lung cancer that was staged separately, the stage in CPFE group was higher compared with the non-CPFE group (*P* < 0.001; Table 5).

**Table 4:** Clinical and pathological results for the comparison groups: lung cancers that developed in a normal lung or a lung with fibrosis or emphysema alone.

Groups	Study/year	No. of pts with lung cancer	Age	Male %	Smoking%	PY	Adeno %	SqCC %	Others %
**Normal**	Fugiwara et al/2013	146	65.6	48.6	71.9	24.4	71.2	13.7	15.1
	Fukui et al/2014	950	65.2	46.1	-	17.1	84.8	8.6	6.5
	Kumagai et al/2014	245	66.0	62.4	41.6	-	91.8	5.7	2.4
	Usui et al/2011	623	66.0	51.5	46.5	5.3	83.0	9.6	7.9
	Pooled Data	1964	65.6	52.0	53.4	15.5	83.8	9.2	7.2
**Fibrosis**	Fugiwara et al/2013	14	72.3	78.6	85.7	36.7	50.0	42.9	7.1
	Fukui et al/2014	84	71.1	66.7	-	29.3	73.8	20.2	6.0
	Kumagai et al/2014	22	75.5	77.3	81.8	-	36.4	45.5	18.2
	Kwak et al/2013	5	66.7^a^	100^a^	76.6^a^	21.2^a^	0.1	60.0	40.0
	Usui et al/2011	15	70.0	13.3	93.3	50.0	46.7	13.3	40.0
	Pooled Data	140	71.7	72.4	84.4	34.0	48.9	32.5	17.8
**Emphysema**	Fugiwara et al/2013	78	66.7	89.7	93.6	51.5	38.5	34.6	26.9
	Fukui et al/2014	197	67.4	87.8	-	55.8	55.8	36.5	7.6
	Kumagai et al/2014	78	70.0	85.9	94.9	-	60.3	33.3	6.4
	Kwak et al/2013	8	66.2^a^	100^a^	97.8^a^	44.5^a^	25.0	62.5	12.5
	Usui et al/2011	404	70.0	85.9	94.6	46.0	48.5	30.9	21.5
	Pooled Data	765	67.8	86.8	94.5	50.1	50.0	33.7	14.1

PY, pack years; Adeno, adenocarcinoma; SqCC, squamous cell carcinoma; pts, patients.

^a^In one study, the value was reported only for the total population, including patients both with and without lung cancer.

**Table 5:** Lung cancer stages in patients with CPFE and non-CPFE.

Stage	CPFE (411/ 413 patients)	Non-CPFE (3598/3633 patients)	*P*-value
**I**	138 (33.4%)	2169 (59.7%)	< 0.001
**II**	74 (17.9%)	435 (12.0%)	
**III**	108 (26.2%)	587 (16.2%)	
**IV**	72 (17.4%)	328 (9.0%)	
**Unknown stage**^a^	2	35	
**Limited disease (SCLC)**	2	9	0.91
**Extensive disease (SCLC)**	17	70	

CPFE, combined pulmonary fibrosis and emphysema; SCLC, small cell lung cancer.

^a^In two studies, pathological staging of the cancers in the indicated patients were unknown [13, 14].

### Outcomes

In four studies which were reported the outcomes of 429 CPFE with lung cancer, 20 (4.7%) patients (6 related to chemotherapy and 14 as postoperative complication) developed acute exacerbation of CPFE [11, 13, 14, 20]. Other postoperative complications such as prolonged air leak, pneumonia, bronchopleural fistula, acute respiratory distress syndrome and empyema were noted in 98 (28.7%) patients according to the three studies which included 341 patients with postoperative outcomes [11, 13, 20].

**Fig 4:**
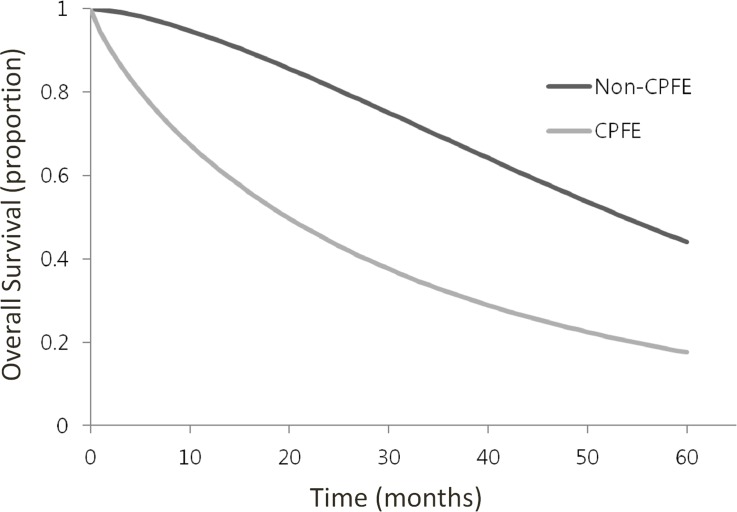
Kaplan–Meier curves for overall survival in lung cancer patients with CPFE versus non-CPRE or normal individuals.

To obtain summary survival curves, 413 lung cancer patients with CPFE and 3633 lung cancer patients with non-CPFE were collected from the 5 studies for which Kaplan–Meier curves for survival analyses were available. To generate summary survival curves, non-CPFE and normal lung groups were combined into a control group and compared with the CPFE group (Fig 4). The median survival time for lung cancer patients with CPFE was 19.5 months, which was shorter than that of lung cancer patients with non-CPFE or a normal lung (53.1 months, *P* <0.001). The 1-, 3-, and 5-year survival rates of lung cancer patients with CPFE were 63.2%, 32.0%, and 17.6%, respectively (Fig 4). In the group of patients with non-CPFE or a normal lung with lung cancer, the 1-, 3-, and 5-year survival rates were 93.1%, 68.5%, and 44.0%, respectively. Patients with CPFE with lung cancer also had lower survival rates compared with the emphysema with lung cancer group.

## Discussion

This meta-analysis involved 620 patients with lung cancer developing in the context of CPFE. The findings suggest that lung cancer in patients with CPFE was most common in elderly heavy smokers with a male predominance, and that squamous cell carcinoma was the most common type of cancer. Compared with the lung cancer population with an otherwise normal lung, the risk of squamous cell carcinoma is 7-fold greater in the CPFE group, and the risk also tends to be higher compared with lung cancers that develop in patients with fibrosis or emphysema alone.

Most of the CPFE patients were smokers and, considering that emphysema and lung fibrosis are tobacco-related diseases, an association may exist with a high risk of developing lung cancer [21]. Squamous carcinoma has been reported to be more significantly associated with tobacco smoking compared with adenocarcinoma, which could be one reason for the greater incidence of this type of cancer in CPFE [22].

Regarding lung cancer staging, the CPFE group included more advanced stages compared with the non-CPFE group (*P* < 0.001). The early detection of lung cancer on chest radiographs can be difficult because tumors may be hidden by the involvement of CPFE. It can also be difficult to identify lung cancer on CT because of concomitant parenchymal fibrosis and emphysema. Thus, a diagnosis of lung cancer may be delayed. Misdiagnoses or delayed diagnoses could be a burden for CPFE patients who have reduced pulmonary function and for the surgeons who had planned to carry out a surgical resection.

The median survival time for lung cancer patients with CPFE was shorter than for lung cancer patients with non-CPFE (*P* <0.001); this finding is consistent with those of previous studies [9–11, 13, 14]. Among the 5 included studies that we used to obtain summary Kaplan-Meier curve data, 2 studies included surgically resected lung cancer patients, so the outcomes may be overestimated. Differences in stages of lung cancer between the CPFE and non-CPFE groups might also affect the survival rates. However, in one study that compared the outcome in the same lung cancer stage patients with or without CPFE, it was found that overall survival was significantly poorer in the CPFE group [13]. Even compared with those lung cancer patients with underlying emphysema or fibrosis, CPFE patients exhibited a higher risk of mortality [9, 10]. Another previous study showed that mortality in patients with CPFE and non-CPFE was similar when lung cancer was not considered as a co-factor [8]. However, mortality in patients with CPFE is affected by coexisting lung cancer. Radiologists should be aware of the risk of lung cancer in CPFE, even though it is not known whether the early detection of lung cancer in CPFE has potential survival gains or not.

This meta-analysis had several limitations, including a heterogeneous study design with various cohort inclusion criteria. Because no consensus definition of CPFE has been widely established, selection bias of reference cohorts and potential diagnosis bias should be considered. Second, the study population in the pooled dataset mainly consisted of Japanese patients, so generalization of these findings might be of limited value. The type of lung cancer in a given country might differ from that of this study; racial differences may represent another concern. Third, because many articles included in this study did not investigate the detailed treatment methods or causes of mortality, this meta-analysis could not focus on these potential variables. Moreover, different distributions of lung cancer stages between CPFE and non-CPFE groups might affect the survival rates. We believe that further studies of CPFE patients with lung cancer will benefit from a consensus of definition, and a prospective evaluation of clinical outcomes with a standard treatment.

In summary, lung cancer in CPFE presented at more advanced stages and had a poor prognosis compared with lung cancer patients with non-CPFE. Additionally, squamous cell carcinoma was found to be the most frequent type of lung cancer in CPFE patients.

## Supporting Information

S1 Fig Summed odds ratio for the proportion of adenocarcinoma in patients with CPFE compared with that in non-CPFE patients. (TIF)

S2 Fig Summed odds ratios for the proportion of adenocarcinoma in patients with CPFE compared with (A) normal lungs or those with (B) fibrosis or (C) emphysema.
(TIF)

S1 File PRISMA checklist. (DOC)

S1 Table Search terms and the number of studies identified from (A) Pubmed, (B) EMBASE and (C) Cochrane Library.
(DOCX)

S2 Table Modified Downs and Black quality scoring system. (DOCX)

S3 Table Results of the risk of bias assessment using the Downs and Black quality assessment scale. (DOCX)
