# Supplementary figures and images for: Lung Cancer in Combined Pulmonary Fibrosis and Emphysema: A Systematic Review and Meta-Analysis
Source: PLoS One. 2016 Sep 12;11(9):e0161437. doi: 10.1371/journal.pone.0161437 (PMC5019377; doi:10.1371/journal.pone.0161437)

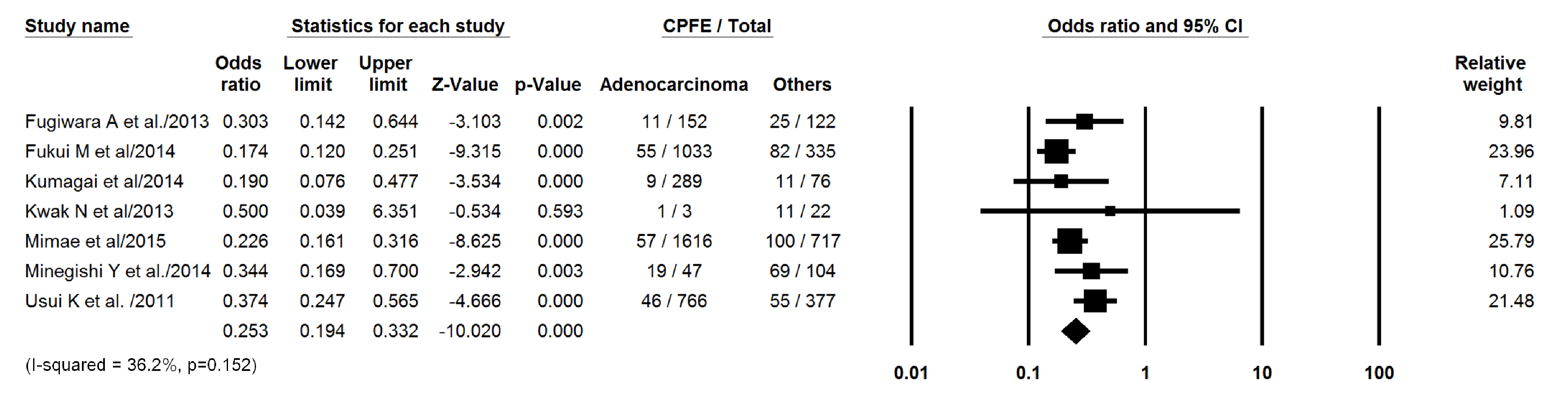

Supplement: S1 Fig — (TIF) [file pone.0161437.s001.tif]

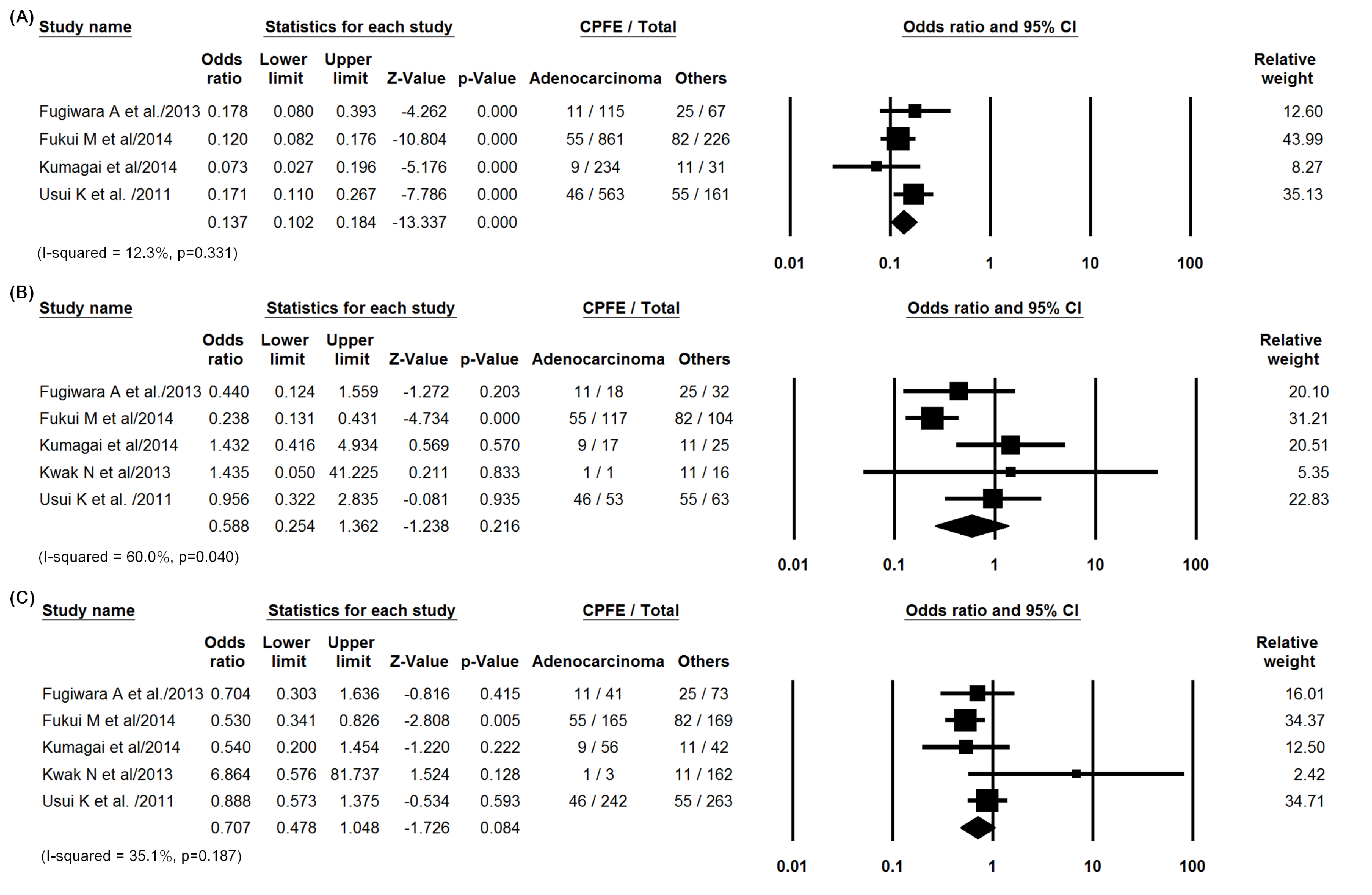

Supplement: S2 Fig — Summed odds ratios for the proportion of adenocarcinoma in patients with CPFE compared with (A) normal lungs or those with (B) fibrosis or (C) emphysema. (TIF) [file pone.0161437.s002.tif]
