# Supplementary material for: Lung Cancer in Combined Pulmonary Fibrosis and Emphysema: A Systematic Review and Meta-Analysis
Source: PLoS One. 2016 Sep 12;11(9):e0161437. doi: 10.1371/journal.pone.0161437 (PMC5019377; doi:10.1371/journal.pone.0161437)
Supplement: S1 Table — Search terms and the number of studies identified from (A) Pubmed, (B) EMBASE and (C) Cochrane Library. (DOCX) [file pone.0161437.s004.docx]

**S1 Table. Search terms and the number of studies identified from (A) Pubmed, (B) EMBASE and (C) Cochrane Library.**

| **(A) Query** | **Results** |
| --- | --- |
| #1 Pulmonary Fibrosis[Mesh] | 17,690 |
| #2 Pulmonary Emphysema[Mesh] | 13,900 |
| #3 #1 AND #2 | 789 |
| #4 Pulmonary[TIAB] OR Lung[TIAB] | 788,858 |
| #5 Fibroses[TIAB] OR Fibrosis[TIAB] OR Fibrosing[TIAB] OR Alveolitis[TIAB] OR Alveolitides[TIAB] | 136,838 |
| #6 Emphysema*[TIAB] | 23,040 |
| #7 #4 AND #5 AND #6 | 1,577 |
| #8 #3 OR #8 | 2,004 |
| #9 (combine* OR cryptogen*) | 834,574 |
| #10 #8 AND #9 | 233 |
| #11 #10 AND ("2005/01/01"[PDAT] : "3000/12/31"[PDAT]) AND English[lang] | 142 |
| Final: (combine* OR cryptogen*) AND ((Pulmonary Fibrosis[Mesh] AND Pulmonary Emphysema[Mesh]) OR ((Pulmonary[TIAB] OR Lung[TIAB]) AND (Fibroses[TIAB] OR Fibrosis[TIAB] OR Fibrosing[TIAB] OR Alveolitis[TIAB] OR Alveolitides[TIAB]) AND Emphysema*[TIAB])) AND ("2005/01/01"[PDAT] : "3000/12/31"[PDAT]) AND English[lang] | |

| **(B) Query** | **Results** |
| --- | --- |
| #1 lung fibrosis'/exp | 57,880 |
| #2 lung emphysema'/exp | 21,357 |
| #3 #1 AND #2 | 1832 |
| #4 Pulmonary:ab,ti OR Lung:ab,ti | 993,277 |
| #5 Fibroses:ab,ti OR Fibrosis:ab,ti OR Fibrosing:ab,ti OR Alveolitis:ab,ti OR Alveolitides:ab,ti | 190,964 |
| #6 Emphysema*:ab,ti | 28,049 |
| #7 #4 AND #5 AND #6 | 2172 |
| #8 #3 OR #7 | 3509 |
| #9 (combine* OR cryptogen*) | 783,318 |
| #10 #8 AND #9 | 394 |
| #11 #10 AND [english]/lim AND [2005-2015]/py | 277 |
| #12 #11 NOT [medline]/lim | 170 |
| **(C) Query** | **Results** |
| #1 MeSH descriptor: [Pulmonary Fibrosis] explode all trees | 217 |
| #2 MeSH descriptor: [Pulmonary Emphysema] explode all trees | 217 |
| #3 #1 and #2 | 3 |
| #4 Pulmonary:ab,ti,kw OR Lung:ab,ti,kw | 47,607 |
| #5 Fibroses:ab,ti,kw or Fibrosis:ab,ti,kw or Fibrosing:ab,ti,kw or Alveolitis:ab,ti,kw or Alveolitides:ab,ti,kw | 6481 |
| #6 Emphysema*:ab,ti,kw | 818 |
| #7 #4 and #5 and #6 | 30 |
| #8 #3 or #7 | 30 |
| #9 #8 Publication Year from 2005 to 2015, in Cochrane Reviews (Reviews and Protocols), Other Reviews and Trials | 10 |
