# Supplementary material for: Lung Cancer in Combined Pulmonary Fibrosis and Emphysema: A Systematic Review and Meta-Analysis
Source: PLoS One. 2016 Sep 12;11(9):e0161437. doi: 10.1371/journal.pone.0161437 (PMC5019377; doi:10.1371/journal.pone.0161437)
Supplement: S2 Table — (DOCX) [file pone.0161437.s005.docx]

**S2 Table. Modified Downs and Black quality scoring system.**

| **Reporting**  1. Is hypothesis/aim/objective clearly described (1)  2. Are the main outcomes to be measured clearly described in the Introduction or Methods section (1)  3. Are the characteristics of the study patients clearly described (1)  4. Are the interventions of interest clearly described (1) 5. Are the distributions of principal confounders in each group of subjects to be compared clearly described (1) 6. Are the main findings clearly described (1) 7. Have all important adverse events that may be a consequence of the intervention been reported (1) 8. Have the characteristics of patients lost to follow-up been described? (OR if none of the participants lost to follow-up, 1) 9. Have actual probability values been reported (e.g., 0.035 rather than <0.05) for the main outcomes except where the probability value is less than 0.001 (1) |
| --- |
| **External validity** 10. Were the subjects asked to participate representative of the entire population from which they were recruited (1) 11. Were those subjects who were prepared to participate representative of the entire population from which they were recruited (1) 12. Were the staff, places, and facilities where the patients were treated, representative of the treatment the majority of patients receive (1) |
| **Internal validity – bias**  13. Was an attempt made to blind study subjects to the intervention they have received (1) 14. Was an attempt made to blind those measuring the main outcomes of the intervention (1) 15. If any of the results of the study were based on “data dredging” was this made clear (1) 16. In trials and cohort studies, do the analyses adjust for different lengths of follow-up of patients, or in case-control studies, is the time period between the intervention and outcome the same for cases and controls (1) 17. Were the statistical tests used to assess the main outcomes appropriate (1) 18. Was compliance with the intervention/s reliable (1) 19. Were the main outcome measures used accurate (valid and reliable) (1) |
| **Internal validity - confounding** 20. Were the patients in different intervention groups (trials and cohort studies) or were the cases and controls (case-control studies) recruited from the same population (1) 21. Were study subjects in different intervention groups (trials and cohort studies) or were the cases and controls (case-control studies) recruited over the same period of time (1) 22. Was there adequate adjustment for confounding in the analyses from which the main findings were drawn (1) 23. Were losses of patients to follow-up taken into account (1) |
| **Power**  24. Did the study have described sample size/ power calculation or included adequate sample size to provide sufficient power to detect a clinically important effect where the probability value for a difference being due to change is less than 5% (1) |
