# Supplementary material for: Lung Cancer in Combined Pulmonary Fibrosis and Emphysema: A Systematic Review and Meta-Analysis
Source: PLoS One. 2016 Sep 12;11(9):e0161437. doi: 10.1371/journal.pone.0161437 (PMC5019377; doi:10.1371/journal.pone.0161437)
Supplement: S3 Table — (DOCX) [file pone.0161437.s006.docx]

**S3 Table.**  **Results of the risk of bias assessment using the Downs and Black quality assessment scale.**

|  | | | | | | |
| --- | --- | --- | --- | --- | --- | --- |
| **Study** | **Reporting (9)** | **External validity (3)** | **Bias (7)** | **Confounding (4)** | **Power (1)** | **Total (24)** |
|  | | | | | | |
| **Kumagai et al/2014** | 9 | 1 | 4 | 3 | 1 | 18 |
| **Fugiwara et al/2013** | 6 | 1 | 2 | 2 | 0 | 11 |
| **Mimae et al/2015** | 9 | 1 | 4 | 3 | 1 | 18 |
| **Minegishi et al/2014** | 9 | 1 | 4 | 3 | 1 | 18 |
| **Usui et al/2011** | 9 | 1 | 4 | 3 | 1 | 18 |
| **Girard et al/2014** | 9 | 0 | 4 | 2 | 0 | 15 |
| **Fukui et al/2014** | 9 | 1 | 4 | 3 | 1 | 18 |
| **Kitaguchi et al/2010** | 8 | 2 | 4 | 2 | 0 | 16 |
| **Kwak et al/2013** | 8 | 1 | 4 | 2 | 0 | 15 |
|  | | | | | | |
